# Shaping the sound of voice

**DOI:** 10.7554/eLife.25858

**Published:** 2017-03-20

**Authors:** Ralph Marcucio

**Affiliations:** Department of Orthopaedic Surgery, University of California San Francisco, San Francisco, United Statesralph.marcucio@ucsf.edu

**Keywords:** larynx, cilia, ciliopathy, CPLANE, Fuz, neural crest, Mouse

## Abstract

The proper development of the vocal cords requires embryos to contain a certain number of progenitor cells, and mutations that lead to an overflow of cells can cause malformations of the voice box.

**Related research article** Tabler JM, Rigney MM, Berman GJ, Gopalakrishnan S, Heude E, Al-Lami HA, Yannakoudakis BZ, Fitch RD, Carter CM, Vokes SA, Liu KJ, Tajbakhsh S, Egnor SER, Wallingford JB. 2017. Cilia-mediated hedgehog signaling controls form and function in the mammalian larynx. *eLife* **6**:e19153. doi: 10.7554/eLife.19153

Acoustic communication is used by many different species. Animals employ sound to attract mates, to sense their environment, to send messages, to convey danger or just to entertain, while insects like ants and crickets also use sound for communication. There is even evidence that some plants use ‘acoustic reflectors’ to attract bats to pollinate, fertilize and distribute seeds (Schoner et al., 2016).

Vertebrates have many different ways to produce sound. Birds sing via a syrinx, for example, while dolphins emit ultrasonic waves by passing air through a structure called the dorsal bursa. Other mammals rely on a complex structure called the larynx that houses the vocal cords and is made of cartilage, muscle, ligament and connective tissue. As the air flows through the larynx, the shape and tension of the vocal cords create sounds through vibration, while the cartilage manipulates the pitch.

Although the biology of language and speech has been studied for decades, our understanding of how the vocal organs develop is still patchy, and most of what we know about the development of the larynx is based on research in bird embryos (Evans and Noden, 2006). As an embryo develops, the cells that will become the vocal organs undergo a series of transformations that are orchestrated by various signaling factors and pathways. Mutations in these pathways can cause structural birth defects, and such mutations may also lead to characteristic vocal traits in humans. For instance, patients with Pallister-Hall Syndrome (Hall et al., 1980), which arises from mutations in a Hedgehog signaling protein called Gli3, are said to have ‘growling’ voices.

Hedgehog signaling occurs within cellular structures called cilia, and patients with mutations in ciliary proteins also suffer from defects in their voice (Beales et al., 1999). It is also known that a ciliary protein called Fuz is required for Gli3 processing (Adler and Wallingford, 2017) but, until recently, it was not known if there was a mechanistic link between disorders affecting the cilia and the development of the larynx. Now, in eLife, John Wallingford and colleagues – including Jacqueline Tabler and Maggie Rigney of the University of Texas at Austin as joint first authors – report that Fuz is essential for the development of the larynx (Tabler et al., 2017).

To better understand how molecular signals and proteins regulate the development of the larynx, Tabler et al. used mutant mice that lacked either Gli3 or Fuz. In both groups of mice, the formation of the larynx was disrupted, but more severely in mice without Fuz. Tabler et al. then looked more closely at proteins of the Hedgehog signaling pathway, which are affected in Fuz mutants. In particular, they focused on a specific type of mutation in the gene for Gli3 that is known to cause birth defects in humans. Indeed, the larynx did not develop properly in these mice because of a build-up of connective tissue near the vocal cords, which affected their ability to make a sound.

In addition, an acoustic map comparing the sounds from wild type and Gli3 mutant mice showed that sound production was negatively affected in the mutants. It appears that changes to the sounds produced are not caused by changes in brain activity, but by physical changes in the larynx itself.

Lying at the heart of the complex process of laryngeal formation and malformation in these mutants is an incredibly simple explanation. Mutant embryos had more progenitor cells – the cells that are destined to build the larynx but have not fully developed yet. Tabler et al. suggest that regulation of the number of progenitor cells could have a role in many disorders affecting the cilia. This is not surprising as the size of the pool of progenitor cells is known to have an important role in other diseases (Jones et al., 2008) and also in evolution (Fish et al., 2014). Some of the observed changes have also been found in other animals, suggesting a conceptual framework for exploring the molecular and developmental basis of evolution that may contribute to diversity of the vocal repertoire among vertebrates.
